# Attitudes and knowledge about naloxone and overdose prevention among detained drug users in Ningbo, China

**DOI:** 10.1186/1747-597X-7-6

**Published:** 2012-02-08

**Authors:** Yu Liu, Nicholas Bartlett, Longhui Li, Xiuyi Lv, Yahai Zhang, Wenhua Zhou

**Affiliations:** 1 School of Medicine, Ningbo University, 818 Fenghua Street, Jiangbei District, Ningbo, Zhejiang 315211, China; 2 Department of Anthropology, History and Social Medicine, University of California, San Francisco, 55 Laguna Street, San Francisco, CA 94102-6232, USA; 3 Laboratory of Behavioral Neuroscience Ningbo Addiction Treatment and Research Center, 42 Xibei Road, Haishu District, Ningbo, Zhejiang 315010, China

**Keywords:** Overdose prevention, Opiate addiction, Naloxone, Peer education compulsory detoxification centers

## Abstract

**Background:**

To date there has been limited research on both the prevalence of overdose and drug user knowledge about overdose prevention and response methods in China. In addition, there has been no effort to integrate naloxone information and distribution into pre-release services for drug users detained in isolated compulsory detoxification facilities in China.

**Methods:**

The authors conducted a survey of 279 heroin users in isolated compulsory detoxification centers in Ningbo, China in an attempt to evaluate the possibility of conducting prelease peer naloxone programs in Ningbo isolated compulsory detoxification centers. Respondents' demographic background, history of heroin overdoses, and attitudes/knowledge about overdose prevention and response were collected.

**Results:**

While drug users in Ningbo's compulsory detoxification centers have limited understandings of how to effectively respond to overdoses, they expressed concern about the possibility of overdose, interest in participating in overdose prevention and response programs, and a willingness to help their peers. In general, there was no significant difference in history and attitudes/knowledge of overdose between male and female participants.

**Conclusion:**

Based on the findings of this research, our survey provides preliminary evidence that detained drug users have considerable interest in overdose prevention and response information and willingness to help peers. However, drug users in Ningbo isolated compulsory detoxification centers currently have limited understandings of effective ways of helping to prevent overdose deaths.

## Background

Overdose is a leading cause of death among illegal opiate users world-wide and has been increasing over the last two decades [1-5]. Drug overdose is also a common cause of non-AIDS death among people with HIV [6]. Naloxone is an opiate antagonist with the property of pharmacologically reversing heroin and other opiate overdoses if administered in a timely manner. The medication has been used for decades by medical professionals [7,8]. Since the mid-1990s, it has also been distributed to drug using peers and other non-medical personnel in an attempt to reduce overdose-related deaths in the community [9,10].

Today, there are hundreds of programs distributing naloxone in over 10 countries around the world [8,11-16]. Evaluations from a number of pilot studies have shown that drug-using peers are capable of effectively administrating naloxone and saving lives [17-21].

Since the late 1980s, illicit drug use and trafficking has been a growing problem in China. The number of registered illicit drug users, mainly heroin users, has increased from 70,000 in 1990 to 1,336,000 in 2009 [22]. Although heroin overdose-related deaths have been reported anecdotally, systematic collection of overdose data in China is still rare in the literature [23]. In a cohort study which enrolled 731 heroin users in Liangshan, Sichuan, China, authors reported a 12% rate of 1-year prevalence of nonfatal overdose and calculated a heroin overdose death rate of 4.7 per 100 persons [24,25]. Heroin overdoses accounted for 68% (30/44) of all deaths reported during the 3-year cohort study [26].

More than half of the registered heroin users reported intravenous injection as their primary mode of heroin administration in China [27]. A number of organizations in China have attempted to integrate overdose prevention and response interventions into harm reduction programming. Daytop, Population Services International (PSI) and various Centers for Disease Control programs in the past several years have offered overdose prevention information in trainings in community peer education programs and in compulsory detoxification-based trainings in Yunnan province. Naloxone has also been widely available in hospitals and emergency ambulances in China and the national methadone maintenance program requests each of the country's over 600 methadone clinics keep the medication on-hand. The first community-based peer administration of naloxone started on a small scale in two sites in 2008. In early 2011, harm reduction programs begun to pilot various models for distributing naloxone in twenty cities in China as part of more robust overdose prevention and response programs [23].

Most of China's isolated compulsory detoxification centers, which housed an estimated 170,000 drug users in 2009, have not integrated overdose response training or naloxone distribution into pre-release programs [28]. To date, there are no published studies examining the knowledge and attitudes of detained drug users towards overdose prevention and response despite the fact that relapse among those released from these facilities is extremely high [29,30]. The present study was conducted to provide evidence about opiate overdose and prevention knowledge among a population of heroin users in Ningbo isolated compulsory detoxification centers.

## Methods

### Participants

In total, 298 patients with a self-reported history of heroin use and a positive opioid urine test prior to entry into the compulsory detoxification center were offered to participate in the study. Research staff ensured that participants understood the decision to participate was voluntary and refusal would not have negative repercussions. A total of 279 patients agreed to complete the study. Before the survey was administered, eligible participants received consent forms distributed by research staff. Anonymity in the data collection process was ensured by not soliciting names or other identifying details on the questionnaire. In addition, after explaining and handing out the survey, researchers and staff members left the room while participants filled out the survey. The participants were asked not to discuss the questionnaires with each other. The study was approved by the Institutional Review Board of the Ningbo Addiction Research and Treatment Center.

### Questionnaire

A brief self-administered, written survey was carried out at the Ningbo Compulsory Detoxification Center in Zhejiang Province. The survey contained 22 questions and required approximately 20 min for participants to fill out. Before the study began, the survey instrument was piloted with 12 methadone clients and subsequently revised to ensure questions were asked in a clear and straightforward manner. Basic demographic information, including gender, age, ethnicity, education, and marital status, was collected. The survey also asked specific questions relating to respondents' heroin use history and experiences of witnessing fatal and non-fatal overdoses. In addition, the survey assessed the respondents' knowledge about heroin overdose, including asking whether respondents believed a range of methods for reversing an overdose were effective. Finally, participants were asked questions about their own likelihood to participate in future efforts to reduce overdoses, including potential interest in attending overdose prevention trainings, as well as willingness to share information and administer naloxone to their peers. Research staff explained to study participants that the definition of an overdose was shallow or no breathing, "pinpoint pupils" or tongue discoloration. Since we were concerned about the educational level of the participants, a simple definition of heroin overdose was used in the present study.

### Data analysis

Demographic and attitude variables were analyzed with descriptive statistics. Pearson *χ*2 or Fisher's exact test was used to examine the differences of history and attitudes/knowledge of overdose between male and female participants when necessary. All tests were two-sided and *p*-value less than 0.05 were considered statistically significant. All statistical analyses were performed using SPSS 10.

## Results

### Demographic information

Participants' demographic information and heroin use history are summarized in Table 1.

**Table 1:** Basic demographic characteristics surveyed patients

	Total	Male	Female
**Number of participants**	279	191 (70.0%)	82 (30%)

**Age (Mean ± STD)**	31.7 ± 7.1	31.3 ± 7.5	32.3 ± 6.2

**Ethnicity**			

**Han**	248 (91.9%)	172 (90.5%)	76 (95.0%)

**Minority**	22 (8.1%)	18 (9.5%)	4 (5.0%)

**Marital Status**			

**Single**	130 (48.9%)	92 (49.7%)	38 (46.9%)

**Married**	92 (34.6%)	71 (38.4%)	21 (25.9%)

**Divorced**	36 (13.5%)	17 (9.2%)	19 (23.5%)

**Separation**	2 (0. 8%)	2 (1.1%)	0

**Widowed**	6 (2.3%)	3 (1.6%)	3 (3.7%)

**Education**			

**Illiterate**	15 (5.7%)	12 (6.5%)	3 (3.7%)

**Elementary school**	72 (27.2%)	53 (28.9%)	19 (23.5%)

**High or vocational school**	124 (46.8%)	80 (43.5%)	44 (54.3%)

**College**	49 (18.5%)	36 (19.6%)	13 (16.0%)

**Graduate school**	5 (1.9%)	3 (1.6%)	2 (2.5%)

**Route of heroin administration**			

**Snorting**	13 (5.4%)	8 (4.7%)	5 (7.2%)

**Smoking**	140 (58.3%)	107 (62.9%)	33 (47.8%)

**Injecting**	55 (22.9%)	34 (20.0%)	20 (29.0%)

**2 or 3 methods**	32 (13.3%)	21 (12.4%)	11 (15.9%)

**Duration of heroin use (years)**	4.82 ± 4.18	4.28 ± 3.67	6.22 ± 5.03

### History of overdose

History of overdose is summarized in Table 2. In total, 104 (37.3%) of the respondents reported having accidently overdosed in the past. Of this group, 75 respondents (72.7%) reported that at least one other person was around them the last time they overdosed. Less than half (39.0%) of respondents reported that someone they knew had died of an overdose. Significantly more female participants reported that someone they knew had died of an overdose than male participants (*p *< 0.05). Of this group of 105 respondents, 18.1% reported having one acquaintance die in the past year, 10.5% reported between two and four, and 2.9% reported having more than ten die within the last year. Of the 37.4% of survey subjects who reported witnessing an overdose, 17.0% reported witnessing one, 9.6% had seen between two and four, 2.1% had seen between five and ten, and 1.1% had witnessed more than ten within the past 12 months. The other person present at this last overdose was most likely a friend who was also using drugs (70.1%). However, family members (17.9%), non-drug using friends (7.5%) and "others" (4.5%) were also reported present at overdoses. The majority of respondents (65.9%) reported that they were concerned about overdosing in the future. However, only 37.2% indicated that they had discussed the topic of overdose prevention and response with their families or friends.

**Table 2:** History of overdose

	Total	Male	Female
**Experiencing heroin overdose**	37.3%	32.1%	48.0%

**Witnessing heroin-overdose**	37.4%	35.6%	41.3%

**Past 12 month, none**	68.1%	66.7%	70.6%

**Past 12 months, 1**	17.0%	15.0%	20.6%

**Past 12 months, 2-4**	9.6%	13.3%	2.9%

**Past 12 months, 5-10**	2.1%	0	0

**Past 12 months, > 10**	1.1	1.7%	5.9%

**Knowing someone who died of heroin overdose**	39.0%	34.4%	49.4%*

**Past 12 months, none**	71.2%	73.2%	67.5%

**Past 12 months, 1**	17.1%	19.7%	12.5%

**Past 12 months, 2-4**	9.0%	7.0%	12.5%

**Past 12 months, 5-10**	0	0	0

**Past 12 months, > 10**	2.7%	0	2.7%

**The other person present at this last overdose**			

**Family member**	17.9%	16.7%	20.0%

**Drug using friends**	70.1%	73.8%	64.0%

**Non-drug users**	7.5%	7.1%	8.0%

**Others**	4.5%	2.4%	8.0%

**Concerned about overdose**	65.9%	68.3%	60.5%

**Discussing overdose with others**	37.2%	33.3%	44.9%

*p < 0.05 a significant difference between male and female participants

**p < 0.01 a significant difference between male and female participants

### Knowledge related to overdose prevention and response

Knowledge related to overdose prevention and response is summarized in Table 3. When asked to choose from a list multiple appropriate ways of responding to a victim of overdose, the most popular answer was "injecting salt water" (56.0%), followed by "calling 120" (emergency) (34.1%), and "pinching an acupuncture point" (21.7%). Only 12.7% of participants selected mouth to mouth resuscitation and 6.0% listed administering naloxone as an appropriate response to an overdose. Significance was found between male and female participants in the following item as a measure to prevent overdose: waking her/him up (*p *< 0.01), pinching an acupuncture point (*p *< 0.01), injecting salt water (*p *< 0.01), drinking water (*p *< 0.05), naloxone (*p *< 0.05), and mouth to mouth resuscitation (*p *< 0.05).

**Table 3:** Knowledge of overdose

	Total	Male	Female
**Signs of heroin overdose**			

**Breathing ↑**	15.8%	15.4%	16.7%

**Breathing ↓**	26.1%	26.5%	25.0%

**Pulse ↑**	20.9%	20.4%	22.2%

**Pulse ↓**	25.2%	24.1%	27.8%

**Pupils ↑**	23.5%	17.9%	36.1%

**Pupils ↓**	10.7%	11.7%	8.3%

**Body temperature ↑**	8.1%	6.2%	12.5%

**Body temperature ↓**	6.8%	4.9%	11.1%

**Loss of consciousness**	51.3%	43.8%	68.1%

**Excited**	7.3%	7.4%	6.9%

**Don't know**	9.4%	11.1%	5.5%

**Responding to a victim of overdose**			

**Waking him/her up**	22.6%	13.1%	44.2%**

**Pinching an acupuncture point**	21.7%	25.1%	46.8%**

**Cold water**	7.7%	5.7%	10.4%

**Injecting salt water**	56.0%	47.4%	75.3%**

**Calling 120 (emergency)**	34.1%	34.9%	32.5%

**Drinking water**	3.2%	2.3%	5.2%*

**Naloxone**	6.0%	7.4%	2.6%

**Mouth to mouth resuscitation**	12.7%	9.7%	32.5%*

**Waking up on his/her own**	2.0%	2.3%	1.3%

**Don't know**	3.6%	4.0%	2.6%

**Injecting salt water is effective to prevent overdose**			

**Yes**	63.4%	60.7%	69.7%

**No**	8.8%	10.4%	5.3%

**Don't know**	27.8%	28.9%	25.0%

**Naloxone is effective to prevent overdose**			

**Yes**	28.2%	32.2%	19.5%*

**No**	8.1%	7.6%	9.1%

**Don't know**	63.7%	60.2%	71.4%

**Having learned cardiopulmonary resuscitation techniques**			

**Yes**	17.6%	19.6%	12.9%

**No**	82.4%	80.4%	87.1%

**Causes of overdose**			

**After drinking heavily**	21.9%	19.0%	27.8%

**Poly-drug use**	14.6%	12.5%	19.0%

**Abstinence**	26.7%	17.3%	46.8%**

**Good quality of heroin**	76.1%	73.8%	81.0%

**Unknown substance in heroin**	18.2%	14.3%	26.6%*

**Don't know**	4.5%	5.4%	2.5%

**How did participants obtain information about heroin overdose?**			

**Friends**	63.1%	56.1%	78.2%**

**Brochures**	23.7%	22.8%	25.6%

**Media**	24.5%	28.1%	16.7%

**Training programs**	3.6%	5.3%	0

**Don't know**	4.8%	5.8%	2.6%*

*p < 0.05 a significant difference between male and female participants

**p < 0.01 a significant difference between male and female participants

When a follow-up question asked explicitly whether injecting salt water was an effective response to opiate overdose, 63.4% responded that it was effective, 8.8% said it was not effective, and 27.8% reported that they did not know. When asked the same question about naloxone, 28.2% said administrating the drug was an effective way to respond to an overdose, 8.8% said it was ineffective, and 63.7% responded that they did not know. Significantly more male users considered naloxone as an effective approach to prevent overdose, compared with female ones (*p *< 0.05).

The vast majority (82.4%) reported they had not learned cardiopulmonary resuscitation techniques in the past. Significance was found between male and female participants in the following item, abstinence as the cause of overdose and "don't know" how to obtain overdose information. More specifically, significantly more female drug users reported abstinence (*p *< 0.01) and unknown substance in heroin as the cause of overdose (*p *< 0.05), compared with male ones. Also, significantly more female users obtained information heroin overdose from friends, relative to male participants (*p *< 0.01).

### Needs relating to overdose prevention and response

When asked whether or not they would be interested in attending trainings on overdose prevention and response if they were offered, 64.0% of participants reported they would be interested. In total, 88.1% of participants reported that they would be willing to administer a medication to an acquaintance having an overdose. Finally, the great majority (69.2%) of respondents reported they would be willing to pass on overdose-related information to their peers.

## Discussion

Research in other countries has shown that individuals recently released from prisons who use opiates are at elevated risk to experience fatal and non-fatal heroin overdoses compared with active opiate users [31-35]. The WHO has recommended that pre-release overdose prevention, including the use of naloxone for those released drug users and others in their social networks [36].

Results from this survey support the idea that overdose prevention training, including naloxone distribution, is needed and wanted in the region. A high percentage of surveyed heroin users lack accurate information related to overdose response. In particular, participants choose "injecting salt water", a strategy that has been proven to be ineffective [37], as an effective way to respond to an overdose at a rate more than one and a half times higher than calling 120 (emergency), five times higher than the rate of performing mouth to mouth resuscitation, and nine times higher than administering naloxone. That accurate information about overdose response is lacking among many heroin users in Ningbo is not surprising given that to date there have been no peer-focused harm reduction activities, including overdose prevention intervention, in the region.

The survey data also shows that there is an interest in overdose prevention and response interventions among detained drug users. Between 64% and 88% of participants in the survey indicated that overdose was a concern in their own lives and, that they would be willing to attend a training program on overdose prevention, help peers administer naloxone, and share information they learned about these services. This finding mirrors international studies that have found that most drug users are willing to help peers [38,39].

Approximately a third of Ningbo respondents reported having accidently overdosed in the past, 37.7% witnessed an overdose, and close to 40% reported having someone who they knew die of an overdose. These numbers are lower than drug user reports of witnessing an overdose in San Francisco (89%) [40], sixteen cities throughout Russian federation (81%) [5], and London (81%) [41]. In Gejiu, China, approximately 90% of drug users surveyed had witnessed overdoses (unpublished data, author communication). In Kunming, China, 34% of respondents recruited from a methadone maintain clinic recently reported being at the site of an overdose of an acquaintance within the last year, nearly three times the number of our Ningbo participants (11.9%) (personal communication, unpublished data). There are a number of factors that may account for the relatively lower occurrence of overdose in Ningbo, such as lower proportion of opiate users injecting (less than 37% of those surveyed indicated that they injected), less mixing of drugs, and relatively low purity of heroin in the region. This underscores the fact that while there is a definite need for overdose prevention and response in Ningbo, there may be even greater need in other parts of the country, where a variety of factors may result in higher number of overdoses.

There are several limitations of this study's methodology that need to be addressed here. Generalization of these results must be made with caution as Ningbo's heroin using population and drug treatment services may not be representative of compulsory detoxification centers in other parts of the country. In addition, the study did not attempt to collect more details of participants' drug use history, information about poly-drug use, or other risk factors that could better identify the specific risk factors associated with overdose among this population. Furthermore, a simple and broad definition of heroin overdose was used in the questionnaire, due to the educational level of the participants. It is possible that this definition of overdose might bias the prevalence estimated here. In the future, more detailed research is needed to better understand what are the risk factors associated with heroin overdoses in Ningbo, where overdose deaths are occurring, and which demographics of heroin users are currently at highest risk. Future studies should recruit in multiple settings to attempt to recruit a more representative sample of those at risk of overdosing.

Despite these limitations, our survey provides preliminary evidence that detaind drug users in Ningbo have considerable interest in overdose prevention and response information and willingness to help peers. However, detained drug users in Ningbo isolated compulsory detoxification centers currently have limited understandings of effective ways of helping to prevent overdose deaths. According to the recent China Drug Control Law, drug dependent patients are required to receive isolated compulsory detoxification for between one and 3 years [42]. Our study indicates that survey respondents had rather limited amount of overdose prevention related information. It should also be noted that relapse rates among individuals released from the isolated compulsory detoxification centers have been found to be extremely high [29,30]. Taken together, we believe that Ningbo isolated compulsory detoxification centers could adopt an overdose prevention and response curriculum in compulsory detoxification centers, including pre-release coordination of prelease distribution of naloxone.

## Competing interests

The authors declare that they have no competing interests.

## Authors' contributions

YL, LL and WZ designed the study. YL, LL, YZ carried ot the study. LL and XL conducted the statistical analysis. YL, NB, and WZ wrote the manuscript. All authors read and approved the final manuscript.

## References

[B1] Bernstein KT Bucciarelli A Piper TM Gross C Tardiff K Galea S Cocaine- and opiate-related fatal overdose in New York City, 1990-2000 BMC Publ Health 2007 7 31 10.1186/1471-2458-7-31 PMC1839087 17349051

[B2] Davidson PJ McLean RL Kral AH Gleghorn AA Edlin BR Moss AR Fatal heroin-related overdose in San Francisco, 1997-2000: a case for targeted intervention J Urban Health 2003 80 2 261 10.1093/jurban/jtg029 12791802 PMC3456286

[B3] Engstrom A Adamsson C Allebeck P Rydberg U Mortality in patients with substance abuse: a follow-up in Stockholm County, 1973-1984 Intern J Addict 1991 26 1 91 10.3109/10826089109056241 2066174

[B4] Hser YI Hoffman V Grella CE Anglin MD A 33-year follow-up of narcotics addicts Arch Gen Psychiatry 2001 58 5 503 10.1001/archpsyc.58.5.503 11343531

[B5] Sergeev B Karpets A Sarang A Tikhonov M Prevalence and circumstances of opiate overdose among injection drug users in the Russian Federation Journal of Urban Health 2003 80 2 212 10.1093/jurban/jtg024 12791797 PMC3456284

[B6] Green TC McGowan SK Yokell MA Pouget ER Rich JD HIV infection and risk of overdose: a systematic review and meta-analysis AIDS 2011 in press 10.1097/QAD.0b013e32834f19b6 PMC3329893 22112599

[B7] Chamberlain JM Klein BL A comprehensive review of naloxone for the emergency physician Am J Emerg Med 1994 12 6 650 10.1016/0735-6757(94)90033-7 7945608

[B8] Kim D Irwin KS Khoshnood K Expanded access to naloxone: options for critical response to the epidemic of opioid overdose mortality Am J Public Health 2009 99 3 402 10.2105/AJPH.2008.136937 19150908 PMC2661437

[B9] Maxwell S Bigg D Stanczykiewicz K Carlberg-Racich S Prescribing naloxone to actively injecting heroin users: a program to reduce heroin overdose deaths J Addict Dis 2006 25 3 89 10.1300/J069v25n03_11 16956873

[B10] Seal KH Thawley R Gee L Bamberger J Kral AH Ciccarone D Naloxone distribution and cardiopulmonary resuscitation training for injection drug users to prevent heroin overdose death: a pilot intervention study J Urban Health 2005 82 2 303 10.1093/jurban/jti053 15872192 PMC2570543

[B11] Buajordet I Naess AC Jacobsen D Brørs O Adverse events after naloxone treatment of episodes of suspected acute opioid overdose Eur J Emerg Med 2004 11 1 19 10.1097/00063110-200402000-00004 15167188

[B12] Coffin P Sherman S Curtis M Cooke K Underestimated and overlooked: a global review of drug overdose and overdose prevention Global State of Harm Reduction 2010: Key Issues for Broadening the Response International Harm Reduction Association 113 119

[B13] Darke S Williamson A Ross J Mills KL Havard A Teesson M Patterns of nonfatal heroin overdose over a 3-year period: findings from the Australian treatment outcome study J Urban Health 2007 84 2 283 10.1007/s11524-006-9156-0 17265131 PMC2231629

[B14] Rosenberg H Melville J McLean PC Acceptability and availability of pharmacological interventions for substance misuse by British NHS treatment services Addiction 2002 97 1 59 10.1046/j.1360-0443.2002.00059.x 11895271

[B15] Thurmond P Bowman S An overview of heroin overdose prevention in the northeast: new opportunities Medicine & Health Rhode Island 2007 90 5 148 17557658

[B16] Wright H Oldham N Francis K Jones L Homeless drug users' awareness and risk perception of peer "Take Home Naloxone" use - a qualitative study Subst Abus Treat Prev Policy 2006 1 28 10.1186/1747-597X-1-28 PMC1599711 17014725

[B17] Lenton SR Dietze PM Degenhardt L Darke S Butler TG Naloxone for administration by peers in cases of heroin overdose Med J Australia 2009 191 8 469 19835548 10.5694/j.1326-5377.2009.tb02891.x

[B18] Piper TM Rudenstine S Stancliff S Sherman S Nandi V Clear A Overdose prevention for injection drug users: lessons learned from naloxone training and distribution programs in New York City Harm Reduct J 2007 4 3 10.1186/1477-7517-4-3 17254345 PMC1797013

[B19] Sherman SG Gann DS Tobin KE Latkin CA Welsh C Bielenson P "The life they save may be mine": diffusion of overdose prevention information from a city sponsored programme Int J Drug Policy 2009 20 2 137 10.1016/j.drugpo.2008.02.004 18502635

[B20] Tobin KE Sherman SG Beilenson P Welsh C Latkin CA Evaluation of the Staying Alive programme: training injection drug users to properly administer naloxone and save lives Int J Drug Policy 2009 20 2 131 10.1016/j.drugpo.2008.03.002 18434126

[B21] Wagner KD Valente TW Casanova M Partovi SM Mendenhall BM Hundley JH Evaluation of an overdose prevention and response training programme for injection drug users in the Skid Row area of Los Angeles, CA Int J Drug Policy 2010 21 3 186 10.1016/j.drugpo.2009.01.003 19268564 PMC4291458

[B22] UNAIDS China Country Office Compulsory and Isolated Drug Rehabilitation Center in China. Situation and opportunities. Presentation at the UNODC & UNAIDS Informal Consultation on Compulsory Centres for Drug Users 2010 Bangkok, Thailand

[B23] Bartlett N Xin D Zhang H Huang K A qualitative evaluation of a peer-implemented overdose response pilot project in Gejiu, China Int J Drug Policy 2011 22 4 301 10.1016/j.drugpo.2011.04.005 21658931

[B24] Yin L Qin GM Ruan YH Qian HZ Hao C Xie LZ Chen KL Zhang YF Xia YW Wu JL Lai SH Shao YM Nonfatal overdose among heroin users in southwestern China American J Drug Alcohol Abuse 2007 38 505 10.1080/00952990701407223 17668336

[B25] Zhang L Ruan YH Jiang ZQ Yang ZN Liu SZ Zhou F He YX Yin L Qin GM Shao YM An 1-year prospective cohort study on mortality of injecting drug users Chinese J Epidemiol 2005 26 190 15941506

[B26] Zhu Y Yin L Ruan Y Hao C Yao H Yang X Bian K Liu L Feng C Qin G Shao YM A 3-Year Prospective Cohort Study on Mortality among Injecting Drug Users in Xichang County (in Chinese) Chinese J Nat Med 2006 8 170 174

[B27] Lu L Fang Y Wang X Drug Abuse in China: Past Present and Future Cell Mol Neurobiol 2008 28 479 10.1007/s10571-007-9225-2 17990098 PMC11515005

[B28] Annual Report on drug Control in China, 2010 Office of China National Narcotic Control Commission 2010

[B29] Sun B Ye Y Qin L An analysis of relapse factors of 615 heroin addicts Chinese Journal of Drug Dependence 2001 10 3 214 216

[B30] Zhao M Hao W yang D Zhang YL Li L A prospective study of factors related to relapse in heroin addicts Chinese Journal of Drug Dependence 2001 9 2 81 84

[B31] Bird SM Hutchinson SJ Male drugs-related deaths in the fortnight after release from prison: Scotland, 1996-99 Addiction 2003 98 2 185 10.1046/j.1360-0443.2003.00264.x 12534423

[B32] Joukamaa M The mortality of released Finnish prisoners; a 7 year follow-up study of the WATTU project Forensic Sci Int 1998 96 1 11 10.1016/S0379-0738(98)00098-X 9800361

[B33] Seaman SR Brettle RP Gore SM Mortality from overdose among injecting drug users recently released from prison: database linkage study BMJ 1998 316 7129 426 10.1136/bmj.316.7129.426 9492665 PMC2665604

[B34] Verger P Rotily M Prudhomme J Bird S High mortality rates among inmates during the year following their discharge from a French prison J Forensic Sci 2003 48 3 614 12762532

[B35] Wakeman SE Bowman SE McKenzie M Jeronimo A Rich JD Preventing death among the recently incarcerated: an argument for naloxone prescription before release J Addict Dis 2009 28 2 124 10.1080/10550880902772423 19340674 PMC2851239

[B36] Prisons and the Health Key Recommendations 2010 WHO

[B37] Curtis M Guterman L Overdose Prevention and Response: A Guide for People who use Drugs and Harm Reduction Staff in Eastern Europe and Central Asia New York: Open Society Institute

[B38] Lagu T Anderson BJ Stein M Overdoses among friends: drug users are willing to administer naloxone to others J Subst Abus Treat 2006 30 2 129 10.1016/j.jsat.2005.05.010 16490676

[B39] Strang J Powis B Best D Vingoe L Griffiths P Taylor C Preventing opiate overdose fatalities with take-home naloxone: pre-launch study of possible impact and acceptability Addiction 1999 94 2 199 10.1046/j.1360-0443.1999.9421993.x 10396785

[B40] Seal KH Kral AH Gee L Moore LD Bluthenthal RN Lorvick J Predictors and prevention of nonfatal overdose among street-recruited injection heroin users in the San Francisco Bay Area, 1998-1999 Am J Public Health 2001 91 11 1842 10.2105/AJPH.91.11.1842 11684613 PMC1446888

[B41] Strang J Best D Man L Noble A Gossop M Peer-initiated overdose resuscitation: fellow drug users could be mobilized to implement resuscitation Int J Drug Policy 2000 11 6 437 10.1016/S0955-3959(00)00070-0 11099924

[B42] Liu Y Liang J Zhao C Zhou W Looking for a solution for drug addiction in China: exploring the challenges and opportunities in the way of China's new Drug Control Law Int J Drug Policy 2010 21 3 149 10.1016/j.drugpo.2009.10.002 19896818

